# Analyzing the landscape of “clean” products for textured hair at a Los Angeles Target

**DOI:** 10.1038/s41370-026-00867-6

**Published:** 2026-04-21

**Authors:** Joaquín Madrid Larrañaga, Lariah Edwards, Robin E. Dodson, Ami R. Zota, Tianna Shaw Wakeman, Janette Robinson-Flint, Bhavna Shamasunder

**Affiliations:** 1 Environmental Studies Program, University of California, Santa Barbara, CA USA; 2 Department of Urban and Environmental Policy, Occidental College, Los Angeles, CA USA; 3 Department of Environmental Health Sciences, Mailman School of Public Health, Columbia University, New York, NY USA; 4 Silent Spring Institute, Newton, MA USA; 5 Black Women for Wellness, Los Angeles, CA USA

**Keywords:** Textured haircare products, Target Corporation, Clean beauty products, Product marketing, Endocrine disrupting chemicals, Web-scraping, Ingredients

## Abstract

**Background:**

Chemicals in haircare products have been linked with endocrine disruption, reproductive health harm, and carcinogenicity. As consumers transition to less chemically intensive hairstyles, they may seek out the “clean” hair product landscape. While large retailers such as Target have created “clean” branded sections to potentially improve transparency for consumers in a complex marketplace, there are no regulatory guidelines defining “clean” and the “clean” haircare market has not been systematically assessed for potential health and safety concerns.

**Objective:**

To assess ingredient hazards within the “clean” textured (curly, wavy, coily) haircare product landscape.

**Methods:**

We web-scraped ingredient lists and other associated information for 150 products for textured hair from the “clean” category on the website for a specific Target store in South Los Angeles. We screened the ingredients for 18 chemicals of health concern (e.g., fragrance, phthalates) and linked the products to the Environmental Working Group’s (EWG) Skin Deep® database to determine a product hazard score (1=least hazardous, 10=most hazardous).

**Results:**

Seventy percent of products listed fragrance, an ingredient category of concern given limited ingredient transparency. Only 62 (41%) of products were listed in the EWG’s Skin Deep® beauty product catalog and over 90% of listed products were classified by EWG as a ‘moderate’ risk to human health (product hazard scores between 3 and 6).

**Significance:**

These findings suggest that people who seek to manage natural hairstyles while avoiding exposure to harmful chemicals, navigate a confusing and unregulated marketplace. Inadequate federal regulation ensuring safety of personal care products does not ensure the safety of products in newer markets of “clean” branded products. Our findings suggest that harmonization of a definition of “clean” should be integrated across industry, from manufacturing to retail given existing inconsistencies that can create challenges for consumers who try to avoid harmful ingredients.

**Impact:**

There is a growing market of “clean” branded beauty products such as Target’s “clean” beauty lines, which market products that exclude ingredients of concern, and thus potentially increase transparency for consumers. By analyzing 150 products for textured haircare found online at a Target store in Los Angeles and by linking products to EWG’s Skin Deep® database hazard scores, we find limitations of such labeling schemes, including the continued existence of hazardous chemicals, potentially misleading marketing language, some inconsistent labeling, and potential negative impacts linked to racial and ethnic identities. Our findings underscore the need for consistency and transparency in labeling products as “clean” to reduce consumer uncertainty and improve product safety.

## Introduction

Consumer products sold in the United States are largely underregulated and are not thoroughly tested or pre-screened before consumer sales [1]. This includes the haircare product industry (classified by the US Food and Drug Administration (FDA) as cosmetics), from salons to home haircare, which is estimated to be a $90 billion market and growing [2, 3]. Prior to 2022, cosmetic companies were not required to report adverse reactions to use of their products and the FDA had no authority to ensure compliance with issued recalls [4]. The Modernization of Cosmetics Regulation Act of 2022 (MoCRA) gave the FDA the power to recall products and now requires companies to disclose ingredients directly to the FDA [4]. However, federal regulation of hazardous chemicals used in cosmetics remains limited [4–6]. State governments have worked to fill these regulatory gaps. For example, the state of Washington recently passed the Toxic Free Cosmetics Act, which restricts certain toxic chemicals and chemical classes from use in cosmetic products manufactured, distributed, and sold in Washington state [7]. In California, the Safe Cosmetics Act of 2005 required cosmetics manufacturers to label any ingredient on state or federal lists of chemicals that cause cancer or birth defects [8]. California regularly updates its reportable ingredients list.

Thus, consumers may be exposed to harmful ingredients with documented adverse effects to humans and must navigate an unwieldy and non-transparent market in an effort to avoid exposures. This effort remains challenging but can be supported by use of publicly available online tools and databases like Environmental Working Group’s (EWG) Skin Deep® database. While this tool aims to provide consumer education and transparency, the scope of available products remains limited in scope and scale, particularly for products used by communities of color, which are often not represented among the available inventories [9]. A study in Boston communities found inequities and higher (more dangerous) Skin Deep® hazard scores in hair products stocked in stores, including Target Stores, located in low-income communities of color [9]. Other studies [10, 11] have utilized the EWG Skin Deep® database to analyze hair product safety, which can help contextualize a given product landscape.

We apply an “environmental injustice of beauty” framework, which highlights intersectional systems of oppression (e.g., racism, sexism, and classism) to unequal chemical exposures, adverse health outcomes, and racialized beauty practices. This framework builds on environmental justice scholarship to further expand environmental racism to include beauty product exposures [12]. Natural hair discrimination, or prejudice against natural hair styles and textures such as locs, braids, fades, and afros is one example of the environmental injustice of beauty. Black women have been pressured to chemically straighten their naturally curly or kinky hair for reasons such as social acceptance or being seen as more professional in the workplace. However, growing movements for women of color aim to embrace more “natural” hairstyles and move away from chemical straighteners [13]. Additionally, an interest in reducing use of chemically intensive products, health concerns, and preference for self-directed hair grooming are reasons women may seek natural hair styles [14, 15] and decision-making could include a range of factors such as costs of chemical treatments, growing health concerns over chemical straighteners, and increased awareness of racialized beauty norms that have promoted straighter hair [12, 13]. Haircare products for textured hair (naturally wavy, curly, or coily hair) include specialized shampoos, hair masques, and curl creams, and are a growing market despite some consumers returning to relaxers due to the time and expense of maintaining natural hairstyles [16].

Chemicals such as formaldehyde, formaldehyde releasers, parabens, phenols, phthalates, and cyclosiloxanes are routinely identified in hair products and length of exposure can directly impact health endpoints [17–19]. These chemicals have been linked with breast cancer, reproductive and metabolic health harm, endocrine disruption, carcinogenicity, and other hormonally related illnesses [18, 20–23], with Black women bearing a differential burden of these illnesses [24]. Many studies have analyzed the harmful effects of beauty products used by Black women [25–28], and as some consumers have sought safer products, there has been limited analysis of the newer “clean” beauty product markets.

### Community-academic research on consumer product use: the taking stock study

This study is part of an ongoing larger community-academic research study: the Taking Stock Study (TSS). TSS is a partnership among University of California, Santa Barbara, Black Women for Wellness (BWW), local promotores de salud (community health workers), Silent Spring Institute, and Columbia University Mailman School of Public Health [29]. TSS was launched in 2018 to understand the role of consumer product use and exposures as an under-examined dimension of environmental justice.

The central neighborhoods for the TSS are situated in South Los Angeles (LA), populated by predominantly low-income Black and Latinx families. Over 90% of residents are people of color (self-identify as Latinx/Hispanic, Black, Asian and/or a race other than White) and approximately three-quarters of residents live below 200% of the poverty line [30]. According to CalEnviroScreen, California’s environmental justice screening tool to identify highly vulnerable communities, South LA is among the top 10% most disproportionately environmentally burdened neighborhoods in the state [31]. The geographic location of the TSS, its retail landscape, and shopping preferences stated by participants in TSS form the basis of this research study, details of which have been reported elsewhere [29].

### “Clean” beauty products

Increasing consumer concerns and growing scientific data about potential exposures from hair product use have pressured the hair sector to offer safer and healthier products [32]. The manufacturing and retail sectors have responded with the creation of new labeling schemes such as “Clean Beauty”, that aim to signal improved, safer, and less toxic product formulations [33]. Additionally, more brands and stores have been selling products that contain “NO labeling” which we define as language that highlights the absence of a specific chemical or ingredient such as “NO artificial colors”, “phthalate free”, and “sulfate free” [34]. These labels may aim to signal to consumers that certain ingredients of concern have been removed from the product. However, these labels are unregulated, and companies may still use these terms even if product formulations never contained these ingredients in the first place. Ingredients such as phthalates or sulfates may be replaced with other, less known, less tested, and potentially dangerous chemicals that may still harm consumers (regrettable substitution) [35]. Target Corporation has been a leader in the sustainability and “clean” beauty space investing over $5 million in green chemistry [36]. Target’s “Clean Beauty” campaign aims to demarcate a category for safer products and may incentivize suppliers to remove ingredients of concern and reformulate their products. Similar programs exist at Sephora, Walmart, and Ulta Beauty. Target has also focused on an appeal to diverse consumers, such as the B3P program and Racial Equity Action & Change program as part of the “Target Forward Vision,” which highlights brands owned by people of color, especially Black and indigenous owned brands and supports those brands to remove harmful chemicals from their products [37]. However, recent shifts in company priorities have since rolled back initiatives to support Black owned beauty brands such as the REACH program [38].

## Methods

We sought to perform a comprehensive assessment based on ingredients listed on the Target website for clean hair products. We focused on products for textured hair because they are marketed to and disproportionately used by women of color, including our TSS study population. First, we identified harmful ingredients in products for textured hair classified as Target Clean. We then used EWG’s Skin Deep® database to obtain hazard scores for these products. We also conducted a case study on a subset of leave-on products to learn more about clean beauty marketing.

### Data source

**Fig. 1:**
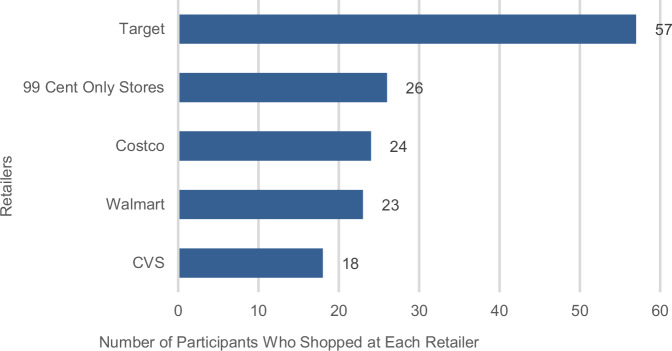
Top five most common retailers (out of 175+ listed retailers) among Taking Stock study participants (*n* = 70).

We focused our analysis on Target because it was the most popular retailer for purchasing personal care products among TSS participants (Fig. 1). Target’s online offerings differ between physical stores based upon products that are available within the physical store or nearby distribution centers. Therefore, we selected a specific Target store in the TSS community.

**Fig. 2:**
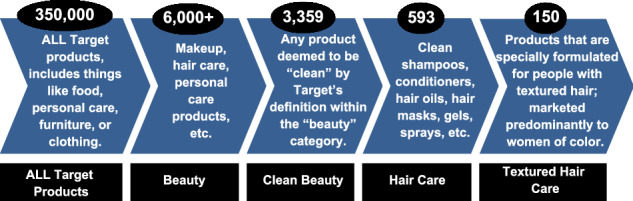
Filtering process for Target Clean textured hair products sold on Target.com for a South Los Angeles store.

We analyzed label information after web scraping textured haircare products sold online by the Target store located at 3535 S La Cienega Blvd, Los Angeles, CA, 90016 that were labeled as Target Clean. The Target.com website uses icons to help consumers seeking clean products. A “Beauty Wellness Icon” signifies that Target has identified a product to be Target Clean, according to their internal definition [39]. We filtered within this larger set of Target Clean products to a subset of products designated by Target that were noted for use by consumers with “textured hair,” a category that is typically marketed towards Black and brown consumers (Fig. 2) [40]. After filtering, 150 products were listed on the Target website that fit these criteria: Target Clean hair products for textured hair.

At the time of data collection, Target defined Target Clean as “products formulated without [<100 ppm] propyl-parabens, butyl-parabens, phthalates, formaldehyde, formaldehyde-donors, nonylphenol ethoxylates (NPEs), oxybenzone, SLES, retinyl palmitate, hydroquinone, triclosan, triclocarbon, and BHA & BHT. Also, cruelty free and/or not tested on animals.” Target now defines Target Clean as not containing any chemicals on the Target Priority Chemical List, a list of 168 chemicals that the Target Company has identified as a high priority for removal [41]. However, this list is not comprehensive so products may still contain regrettable substitutions, and verification, while done by a third party, is not always done via testing and may rely on disclosure. Other icons meant to help consumers include “Formulated without parabens,” “Formulated without Phthalates,” and “Cruelty-Free.”

### Web-Scraping

We scraped the Target.com website in September 2022 using custom python code that employed the Beautiful Soup v4.11.1 text parser Python library and the Selenium Webdriver v4.2.0, an automated web-browser interaction tool [42–44]. The web-scraped data for each product included product title, a description, product specifications (Scent, Hair Type, Health Facts, Product Warnings, Product Form, Recommended Use, Package Quantity, Beauty Purpose, Net weight, TCIN, UPC, Item Number [DPCI], Origin), price, ingredients, product brand, and Target rating out of 5 stars. Each product’s wellness icons (e.g., “Clean,” “Phthalate free,” “Vegan,” or “Cruelty Free”) were also web-scraped. The Target website groups products in sets of 30 products per page. The first product on each product webpage (3% of products) was verified for correctness following the web-scraping. Following data collection, we calculated each product’s unit price (US dollar per fluid ounce) in order to compare prices across products.

### Analyzing product safety via the Environmental Working Group hazard score

The Environmental Working Group’s Skin Deep Cosmetics Database was utilized to identify the product’s hazard score. We first analyzed product ingredient labels for a select number of chemicals of health concern. We then cross-referenced each product name with the Environmental Working Group’s (EWG) Skin Deep® database to determine each product’s hazard score. The Skin Deep® database is an online tool where consumers can look up their products and evaluate their safety using a hazard score that is based on the products’ ingredients [45].

We queried for parabens (EWG score 3–10), sulfates (EWG score 1–3), and phthalates (EWG score 1–10) both because these chemicals are listed in Target’s definition of Clean and because of recent industry efforts to remove these chemicals [21]. We focused on the ingredient terms “fragrance” and “parfum” (EWG score 8) as these are umbrella terms that represent a mixture of undisclosed chemicals, some of which could be linked with health hazards. For example, fragrance ingredients such as alpha-isomethyl ionone, benzyl salicylate, hexyl cinnamaldehyde, linalool and piperonal, among others, have been linked with adverse health effects like allergic reactions, dermatitis, and skin cancer [19, 46–48]. In addition to parabens, we also queried our ingredient data set for two other common preservatives assumed to replace parabens in personal care products: isothiazolinones (EWG score 1-7) and phenoxyethanol (EWG 2-4). Isothiazolinones (e.g. methylchloroisothiazolinone [MCI]) have been shown to cause allergic reactions, dermatitis, and may be a neurotoxic biocide [49–51]. Phenoxyethanol, widely considered to be a less harmful substitute for parabens [52], may still cause skin irritation and contact dermatitis. Phenoxyethanol is regulated by the EU and French authorities to be used only in concentrations under 1% because of concerns to human health [53]. We also queried for formaldehyde (EWG score 10), a known human carcinogen [54], and two of its common synonyms, methylene glycol (EWG score unknown) and formalin (EWG score 9) [19].

The EWG Skin Deep® database includes over 80,000 products from 2500 brands and products in the database are given a “hazard score” on a scale of 1 to 10, with 10 being the most hazardous. These scores are calculated based on factors from nearly 60 integrated toxicity, regulatory, and study availability databases [55]. Information regarding harmful ingredients, product application method, length of exposure, and more from these databases are synthesized and weighted to produce the overall EWG score. Chemical ingredients are weighted according to depth of research regarding toxicity. For example, if an ingredient is a known carcinogen, it is weighted as 100 in the category of carcinogenicity, but if an ingredient is only a probable carcinogen, it is weighted accordingly, i.e. 55. Carcinogenicity is just one of EWG’s 17 weighted toxicity and hazard categories [45]. Scoring in the database is based on the weight of the available scientific evidence, with detailed information on the hazard rating available [55]. Products scoring a 1 or a 2 can also receive an EWG VERIFIED™ mark signaling these products do not use ingredients on EWG’s “unacceptable list” and meet documentation requirements for restricted ingredients [45]. However, products only receive an EWG VERIFIED™ designation after collaborating directly with EWG and EWG will not independently award EWG VERIFIED™ ratings. We collected product hazard scores for each product in July 2023.

### Comparison of product labeling and EWG score

We also conducted a market scan of product brands contained in the scraped dataset. For each brand, we documented any “clean” language featured on the brand’s landing page of their website. We documented the use of words like “clean”, “natural,” or “organic,” that were used to describe the product or the brand overall. “Clean” language also included any mention of excluded ingredients, such as “no parabens,” “phthalate free,” or “formulated with no sulfates” which we called “NO labeling.” We conducted the market scan in June–October 2023.

We also conducted a product case study to investigate trends between product descriptions that contained “clean” language and a product’s overall EWG score. We focused our case study on the two largest categories of leave-in hair products: leave-in conditioners and moisturizers (i.e., products used to moisturize the hair), and hair stylers (e.g., curling creams, gels, hair oils, or sprays) as they have the potential highest exposure due to a high probability of being left on the hair or scalp the longest. We chose 18 products, 9 in each category, with the intention of including a variety of brands and a range of EWG scores. Products were chosen by first prioritizing products with scores at the lowest and highest of our score range of our dataset (1–7) within the two product categories. Since many products had scores of 4 or 5, we then tried to select products from different brands. For each product, we documented “clean” language on the product page on the brand’s website and on the product label. Lastly, we re-engaged EWG’s product database in October of 2024 to determine which ingredients were the biggest drivers of high product scores for products in our case study. We noted dates as product formulations may have changed between the original dataset scraping in 2022 and investigating the ingredients driving higher scores (scores > 2) in 2024.

## Results

### The product landscape of products sold at a South Los Angeles Target with the label “clean”

**Fig. 3:**
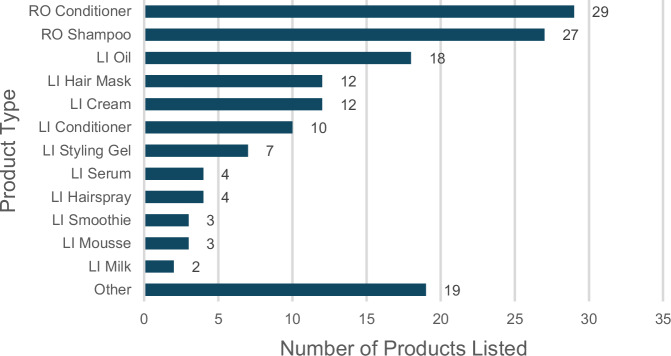
The landscape of 150 Target Clean products for textured hair sold at a South Los Angeles Target. LI indicates “leave-in” products (products that direct users to leave the product in for more than 3 min) and RO indicates “rinse out” products (products that direct users to rinse the product out immediately or before 3 min have elapsed).

The 150 products we included in our analyses represented 28 different brands (Fig. 3). Almost one-fifth of the products were rinse out conditioners. The next biggest category of products was rinse out shampoos followed by leave-in oils. Products were categorized as “leave-in” (54.7% of products) if product instructions directed consumers to leave the product in for more than 10 min. Some products (such as most conditioners) directed users to leave products in for “up to 3 min” but then directed users to “thoroughly rinse” the product out. These products are listed as “rinse out” (39.9%). The category of “Other” (6%) includes 12 distinct categories of products including but not limited to detanglers, butters, infusions, lotions, as well as 6 products that did not provide directions for use on the Target website. Some of these products offered multiple uses (e.g., a conditioner that can be used as either a rinse out or leave-in conditioner) or did not indicate directions for use. The average price per ounce for products analyzed was $1.75.

### Identifying products with specific ingredients of health concern

Of these 150 products, 70% listed “fragrance” or “parfum” as an ingredient. While none of the products contained known parabens on the label, 27 products did not have the “Formulated without Parabens” Target badge. Of the 150 products, 14.6% of products contained sulfates, although only 50.6% of products listed the “Formulated without Sulfates” Target badge, leaving 34.7% of products inconsistently labeled. Two products contained isothiazolinones and 40 products contained phenoxyethanol. None of the products contained any of the chemicals listed in Target’s definition of “clean”.

### EWG safety ratings

**Fig. 4:**
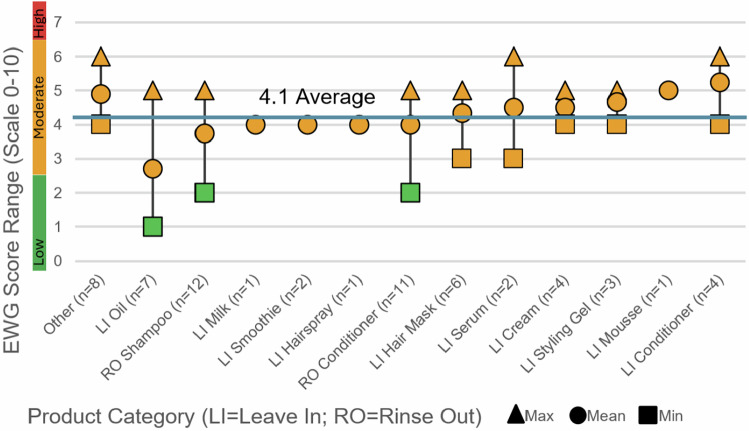
*Hazard scores (scale from 0-10), by product category for the 62 hair products available in the EWG Database.* Maximum, mean, and median scores shown. LI indicates “leave-in” products (products that direct users to leave the product in for more than 3 min) and RO indicates “rinse out” products (products that direct users to rinse the product out immediately or before 3 min have elapsed). Number of products within each category is shown in parentheses.

Only 62 products of the 150 products analyzed (41%) were listed on the EWG database, thus the majority of products (59%) in this category lacked an EWG score. The average EWG score for these 62 products was 4.1 (Fig. 4), which is considered a **moderate hazard** for human health. EWG scores of these 62 products ranged from 1 to 6 with a median score of 4. “Leave-in” conditioners had the highest average EWG scores while “leave-in” oils had the largest range of EWG scores (1–5).

**Fig. 5:**
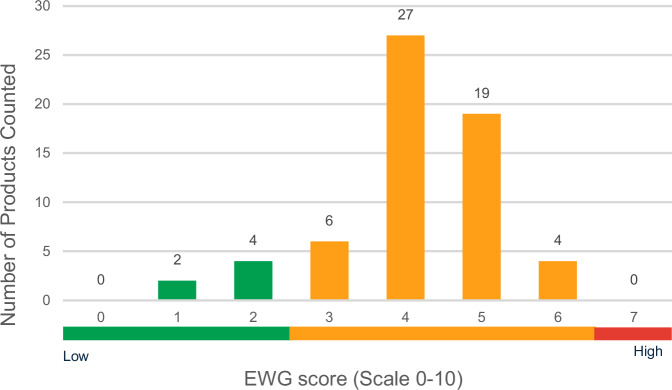
Distribution of EWG scores for 62 hair products. Number above the bar indicates number of products with that score. Color coding matches color coding used in EWG database. Green scores considered “Low Hazard,” orange scores considered “Moderate Hazard,” and red scores considered “High Hazard.

Of the 62 products, only 6 products are rated by EWG as “low hazard” (score <2, Fig. 5). Ninety percent of the 62 products were classified as a moderate hazard to people’s health (scores 3–6). Three products (two with a score of 4 and one with a score of 2) contained ingredients classified by EWG as “possible human carcinogens”. We did not observe a relationship between EWG score and total number of ingredients per product.

### Product marketing and EWG scores

In a smaller case study analysis, we compared “clean” language found in product descriptions and EWG scores for 9 leave-in conditioners and moisturizers and 9 hair stylers. All the leave-in conditioners (9 out of 9) and 77% of the stylers (7 out of 9) evaluated had “clean” language on their product labels (Table 1). At the time of the evaluation, 64% of brands categorized as Target Clean had “clean” language on their brand’s website’s landing page. Most brands emphasized the different chemical ingredients excluded from their products on their landing pages. Similarly, our case study analysis found that most brands emphasized the different chemical ingredients excluded from their product’s formulation on the product labels (a form of “no-labeling”). This language was less commonly featured on a product’s brand page, with 44% of leave-in conditioners and moisturizers (4 out of 9) and 22% (2 out of 9) of hair stylers having language about excluded ingredients on their product pages (Table 1). We did not observe a relationship between EWG score and “clean” language for these 18 products. The presence of “fragrance” on the ingredient list was the driver of many of the highest scores. A majority of our products fell within the moderate range of EWG scoring, 3 to 6, and almost all had some sort of featured “clean” language.

**Table 1:** Comparison of EWG score and presence of “clean” language on products’ labels or web pages for both conditioners & moisturizers, and stylers.

Conditioners and Moisturizers	EWG Score	“Clean” Language on Product Label	“Clean” Language on Product Website	Ingredient with highest hazard score (Determined in October 2024)
Curls Blueberry Bliss Reparative Leave In Conditioner - 8 fl oz	4	No sulfates, silicones, parabens, artificial oils, colors or fragrance	No	Fragrance
Curl Dynasty Give Me Slip Blast Hydration Conditioner - 8 oz	4	Certified organic	No	Fragrance
Pacifica Pineapple Curls Curl Defining Conditioner - 8 fl oz	4	Silicone-free	No	Fragrance
Palmer’s Coconut Oil Formula Moisture Boost Deep Conditioner - 12 oz	5	Free of parabens, phthalates, gluten and dyes	Product pages say “No parabens, phthalates, mineral oil, gluten or dyes”	Fragrance
SheaMoisture Jamaican Black Castor Oil Strengthen & Restore Conditioner - 24 fl oz	5	Sulfate free and color safe	No	Behentrimonium chloride
Wakati Water Activated Conditioner, Paraben and Sulfate-Free for Natural Hair, Easy Finger Tangling - 8 oz	5	Paraben-free, sulfate free	Product pages have badges for paraben free, phthalate free and SLS/SLES free	Fragrance
Curls Lavish Curls Daily Moisturizer - 8 oz	6	No sulfates, silicones, parabens, artificial oils, colors or fragrance	No	^b^
Garnier Fructis Sleek & Shine Smooth Leave-in Conditioning Cream^a^	6	--	No parabens, no phthalates, and no DMDM hydantoin.	Fragrance
Hask Argan Oil Repairing Deep Conditioner - 1.75 fl oz	6	No sulfates, no parabens Hask Clean Beauty	Free of: sulfates, parabens, silicones, phthalates, gluten and drying alcohol.	Fragrance
**Hair Stylers**				
SheaMoisture 100% Pure Argan Oil	1	--	--	Argan oil (the only ingredient)
Alodia Nourish and Grow Healthy Hair and Scalp Oil	2	--	100% Natural ingredients	Sweet basil oil - masking, tonic, fragrance ingredient, skin-conditioning agent
Curls Blueberry Bliss Hair Growth Oil	3	No sulfates, silicones, parabens, artificial oils, colors or fragrances	Ingredients that are organically sourced are denoted	Fragrance
Wakati Oil-Infused Cream	4	Paraben free, SLS/SLES free	Paraben free, SLS/SLES free, phthalate free	Fragrance
Curl Dynasty Twisted Definition Twisting Cream	5		Ingredients that are organically sourced are denoted	Fragrance
Rucker Roots Leave-In Heat Protectant	5	No sulfates, parabens, petroleum’s, artificial color or mineral oil	Natural ingredients are highlighted on the product page, Proudly made without sulfates, parabens, artificial color, petroleum, mineral oil	Fragrance
SheaMoisture Coconut & Hibiscus Curl Enhancing Smoothie	5	No parabens, no phthalates, no propylene glycol, no mineral oil, no animal testing, no petrolatum	--	Fragrance
SheaMoisture Coconut & Hibiscus + Flaxseed Defining Styling Gel	5	Non-alcohol based formula, no parabens, non phthalates, no mineral oil, no animal testing, no petrolatum	--	^b^
TPH Keep Shining Dry Oil Mist	5	Silicones free, SLS/SLES free, phthalate free, paraben free, cruelty free, vegan	No phthalates, No SLS/SLES, no parabens, no mineral	Fragrance

^a^Unable to read the images of the product’s label online.

^b^Product ingredient information no longer available in October 2024.

## Discussion

We analyzed ingredient lists for 150 textured haircare products labeled as Target Clean and sold online at a Target in South Los Angeles. Less than half of 150 textured haircare products categorized as Target Clean were in EWG’s Skin Deep® database, potentially making even this third-party verification tool of more limited usability by consumers who seek products in the textured haircare section. Of products that were found, majority of the products in the Clean database were classified as posing at least moderate hazard to human health. None of the products were ranked as “EWG VERIFIED^TM”^, the highest standard for “clean” given by the non-profit (though we note there is a cost per product associated with becoming “EWG VERIFIED^TM^,” a system that may prevent smaller brands with fewer resources from ever getting verified). A majority of the 150 products included the umbrella ingredient terms “fragrance” or “parfum,” which may include a range of harmful ingredients but do not require ingredients disclosure through “trade secrets” protections [18]. Fragrance ingredients drove higher hazard scores in the EWG database. In an in-depth case analysis of 18 leave-on products, we found that a majority of brands used “NO labeling” to advertise that their products are free of certain chemicals, and by using the word “clean” in their website marketing. However, clean is not always synonymous with “NO labeling”, which could create confusion among consumers. This underscores the limited value of the use of the word “clean” for consumers, since the word is often used with different definitions from one product to another, highlighting a lack of clarity and transparency.

A few other studies have used the EWG Database to evaluate product safety [9–11]. After analyzing cosmetics sold by Sephora, Phan et al. (2021) reported a correlation between EWG score and “clean” (products containing the Sephora Clean seal) versus “not-clean” products with “clean” products having lower scores (average score of 3.1 for clean products and 4.1 for not-clean products). Our research demonstrates that “clean” hair products at Target have an average score of 4.1, a higher average score than Phan et al., suggesting that textured haircare products not identified as “clean” at Target may have even higher average scores, though this comparison was not carried out. Chan et al. (2023) found that hair products available at stores (including Target stores) across 8 different neighborhoods ranging in SES in Boston, Massachusetts had mean EWG scores of 5 to 5.9 finding a two-fold higher risk ratio for products sold in lower income communities of color [9]. An EWG report in 2025 analyzing the EWG database for products marketed to Black women places about 80% of haircare products in the moderate hazard (3–6) range [56]. This is especially concerning considering 90% of products in our analysis score above a 3, refuting the hypothesis that “clean” products would have lower average EWG scores. Thus, when consumers seek safer products, they may still be purchasing products with concerning or unknown chemicals. In addition, the under regulation for the use of a “clean” label potentially implies a level of safety that should be made both clear, well-defined, and transparent to the consumer. Additional research is needed to evaluate safety of “clean” products for other product categories.

We found that only 41% of the 150 hair products analyzed were found in the EWG database. Chan et al. found that in neighborhoods chosen for their high percentages of Black, Asian, and Hispanic/Latina populations, 35% to 94% of hair products surveyed from these neighborhoods did not have an EWG rating. The significant portion of missing products that are used by study participants in both studies highlights a critical data gap for residents in these communities who are trying to navigate the haircare landscape, demonstrating the need for more extensive publicly available databases for consumer safety.

While “clean” labeling schemes aim to help consumers limit chemical exposures, our research demonstrates that there remains limited ingredient transparency due to umbrella terms like “fragrance” and inconsistent corporate labeling. This can create potential confusion among consumers. Efforts to address this include regulatory requirements for consistent labeling for the benefit of consumers and a harmonization of the definition of “Clean” across the industry from manufacturing to retail. Of the 150 products analyzed, 27 products did not have the “Formulated without Parabens” Target badge and 53 did not have the “Formulated without Phthalates” badge despite qualifying for these badges, which adds to the confusion for consumers when labeling is inconsistently applied. This raises several questions about consistency across Target labeling because by definition products in the “clean” category qualify for these badges. Target recognizes that they rely solely on the manufacturer labels for their “Formulated without Parabens” and “Formulated without Phthalates” badges [57]. Thus, efforts to address existing lack of transparency or confusion in labeling must begin at the manufacturer level, especially given ingredients might not appear on the label or be “NO labeled” but could still contain these chemicals [48, 58, 59]. Regulators must also ensure that chemicals chosen to replace hazardous chemicals are low hazard, for example an EWG score of <2. Retailers, such as Target, that create internal definitions for “clean”, should maintain consistency across its application for these efforts to be most beneficial for consumers.

Products marketed as “clean” tend to have a higher purchase price than their counterparts [60]. A previous analysis found that lower-priced products may be more prevalent in lower-SES neighborhoods, but higher-priced products may have lower hazard scores [9]. Fifty-eight percent of our TSS population purchased products online and Amazon was the top online retailer among TSS participants while Target was the top physical retailer. The average price per ounce for the “clean” products for textured hair found online at Target that we analyzed here was $1.75. This price is more than three times more expensive than 20 textured hair products not labeled as clean scanned from Amazon which have an average price per ounce of $0.56 ± $0.053 [61], suggesting that product price may be a barrier for selecting cleaner products. While this study did not compare product prices in the South Los Angeles Target versus other stores, the per ounce comparison to other non “clean” products sold online [61] supports Chan et al. 2023’s findings - “clean” beauty is typically more expensive, which may be out of reach for some consumers. Further, for those who do purchase “clean”, they may be unaware of potentially harmful regrettable substitutions or other potential trade-offs.

### Limitations

Product formulations may have changed since we conducted our analysis in late 2022. Also, as of 2023, Target expanded their Target Priority Chemical List (TPCL), a restricted ingredient list for their products [62]. However, isothiazolinones nor phenoxyethanol, among other potentially hazardous ingredients, were not added to the list, despite emerging concerns for their toxicity. Therefore, ingredients of health concern are still likely found in products across the US due to lack of federal regulation.

Our analysis relied on EWG’s Skin Deep database, a popular tool among consumers seeking to evaluate product ingredients. Although other product screening tools exist (ChemFORWARD, GreenScreen, etc.), these tools are not as widely marketed or built for consumer use. While EWG aims to provide transparency and assist consumer decision making, it does have limitations. For example, EWG assigns “Fragrance” a score of 8 regardless of the constituents [45]. Additionally, chemicals with little or no data may be assigned a low hazard score. To handle limited toxicity information, EWG recommends that users ensure their products also have a high “Data Availability Rating.”

Many products in our analysis were not in the EWG database so were not included in the case study analysis. Furthermore, there was a two-year gap between EWG score used in determining inclusion in the case study and the investigation of the highest rated ingredient. Lastly, for the case study analysis we focused on a subset of products, limited to conditioners, moisturizers, and stylers. As such, our conclusions about language on the label and website as they relate to EWG score may not be generalizable to other products.

## Conclusion

In the absence of federal chemical policy, consumers oftentimes must rely on incomplete manufacturer and retailer provided information to evaluate the safety of their products. As such, the “clean” beauty market has grown, perhaps seeking a marketing advantage [63]. The result is that consumers may be subjected to marketing messages that sell health and safety claims that are not well-substantiated or are confusing. While Target, a popular retailer among our TSS participants, continues to make strides towards stricter chemical safety (incentivizing manufacturers to remove chemicals of concern, investing in research to close data gaps, etc.), we find the “clean beauty” category to be inconsistent.

A harmonized definition of “Clean” across the supply chain from manufacturing through retail should be created, used consistently across the industry, and regulated, for the benefit of consumers seeking safer products. Our data supports the need for increased labeling and marketing transparency from industry to consumers, so consumers can make informed decisions about their product use and, in particular, to support the purchasing of safer and healthier products. In addition, voluntary industry efforts remain limited and assert the need for improved national policy.

We found the “clean beauty” category to be inconsistent. Our analysis supports the need for better and more consistent labeling and marketing transparency from industry to consumers, so consumers can make informed decisions about their product use and, in particular, to support the purchasing of safer and healthier products. In addition, voluntary industry efforts remain limited and, in this context, assert the need for improved national policy.

## Data Availability

The datasets generated during and/or analysed during the current study are available upon request.
